# Long‐term safety of brodalumab in Japanese patients with plaque psoriasis: An open‐label extension study

**DOI:** 10.1111/1346-8138.15343

**Published:** 2020-04-10

**Authors:** Yukie Yamaguchi, Nobumichi Takatsu, Kenji Ootaki, Hidemi Nakagawa

**Affiliations:** ^1^ Department of Environmental Immuno‐Dermatology Yokohama City University Graduate School of Medicine Yokohama Japan; ^2^ Medical Affairs Department Kyowa Kirin Co., Ltd. Tokyo Japan; ^3^ R&D Division Kyowa Kirin Co., Ltd. Tokyo Japan; ^4^ Department of Dermatology The Jikei University School of Medicine Tokyo Japan

**Keywords:** brodalumab, Japan, long‐term care, psoriasis, safety

## Abstract

Brodalumab, an interleukin‐17 receptor A inhibitor, demonstrated rapid and robust efficacy with a favorable safety profile in patients with moderate to severe plaque psoriasis. Here, we present data from a multicenter, open‐label extension study in patients with plaque psoriasis with/without psoriatic arthritis who completed 64 weeks of treatment with brodalumab (140 or 210 mg, every 2 weeks [Q2W]). Patients were enrolled to evaluate the long‐term safety and efficacy of a modified dose of brodalumab. Eligible patients were switched to a reduced dose of brodalumab (140 mg every 4 weeks on day 1) in the extension study; the dose and dosing interval were modified sequentially at the physician’s discretion (minimum 140 mg every 8 weeks and maximum 210 mg Q2W) until drug approval, after which all patients were switched to 210 mg Q2W for postmarketing surveillance. Of the 129 patients enrolled, 107 (82.9%) completed the 108‐week or more extension study. All patients had psoriasis that was well controlled with brodalumab treatment on day 1. Improvement in psoriasis‐related symptoms, evaluated with the Psoriasis Area and Severity Index, Psoriasis Scalp Severity Index, Dermatology Life Quality Index, Nail Psoriasis Severity Index, and American College of Rheumatology 20, 50 and 70, was maintained during the 108‐week extension study. Brodalumab treatment was well tolerated throughout, and no new safety signals were identified. The most commonly reported treatment‐related adverse event was nasopharyngitis, followed by influenza and oral candidiasis. No cases of serious candida infection or Crohn’s disease were observed in this study. Serious treatment‐related adverse events, such as appendicitis, brain abscess, bacterial meningitis, colon cancer, immunoglobulin A nephropathy and tubulointerstitial nephritis, were reported in one patient each. No anti‐brodalumab‐binding antibodies or brodalumab‐neutralizing antibodies were detected in any patient throughout the extension study. Overall, the long‐term efficacy and safety of brodalumab were demonstrated over 108 weeks.

## Introduction

Psoriasis is a systemic chronic inflammatory disease with an estimated global prevalence ranging 0.09–11.4%; the prevalence for all ages was 1.43–5.1% (1971–2009) in the USA^1^, ^2^ and 0.44% (2010–2011) in Japan.^3^, ^4^ Psoriatic patients experience a substantial burden of the disease, with physical and psychological comorbidities that further deteriorate the health‐related quality of life (HRQoL).^5^

As the interleukin (IL)‐17/IL‐23 axis is implicated in the pathogenesis of psoriasis, inhibition of IL‐17 has emerged as an important therapeutic strategy for the management of psoriasis.^6^

Brodalumab, a fully human immunoglobulin (Ig)G2 monoclonal antibody produced in Chinese hamster ovary (CHO) cells, binds selectively to the human IL‐17 receptor A (IL‐17RA) and inhibits the biological activities of IL‐17A, IL‐17C, IL‐17E, IL‐17F and IL‐17A/F heterodimers.^7^, ^8^

Brodalumab is currently approved for the treatment of moderate to severe plaque psoriasis in adult patients^7^, ^8^ and is administrated s.c. at a dose of 210 mg at weeks 0, 1 and 2 of treatment, followed by 210 mg once every 2 weeks (Q2W).^7^ In a 12‐week, phase II, randomized controlled study conducted in Japanese patients with moderate to severe psoriasis, brodalumab demonstrated rapid and robust efficacy with a favorable safety profile (NCT01748539).^9^ It also showed a sustained clinical response with an acceptable safety profile over 52 weeks in Japanese patients with moderate to severe plaque psoriasis in an open‐label phase III study (NCT01782924).^10^ Another phase III study was conducted in Japan to evaluate the efficacy and safety of brodalumab in patients with plaque psoriasis and in patients with pustular psoriasis or psoriatic erythroderma (NCT01782937).^11^ This study was an extension of the above two phase III studies.

The objectives of the present analysis were to evaluate the long‐term safety and efficacy of brodalumab in patients with plaque psoriasis except in those with pustular psoriasis and psoriatic erythroderma.

## Methods

### Study design

This long‐term, multicenter (54 study sites), open‐label extension (OLE) study (NCT02052609^12^) was tandemly conducted in patients with plaque psoriasis after the phase III study.^10^ This OLE study converted into the post‐approval phase study (NCT04183881^13^; 4 July 2016 to 31 December 2016) after brodalumab was approved for use in Japan (Fig. S1). Here, we present the data of patients with plaque psoriasis who were enrolled in this extension phase after they completed the 12/52 weeks of treatment in the phase II and phase III studies^9^, ^10^ of brodalumab in Japan.

Eligible patients with plaque psoriasis were treated with s.c. brodalumab at a dose of 140 mg on day 1 and 140 mg once every 4 weeks (Q4W) thereafter. The dose and dosing interval were modified at the physician’s discretion, if needed, based on specific criteria (mentioned below) until the study was converted to a PMS study, after which all patients were switched to receive brodalumab 210 mg Q2W.

Overall, patients were followed up on day 1 (week 0) and Q2W thereafter until the end of the study (EOS). EOS was defined as either treatment discontinuation or the date of study completion. After enrollment (week 0), patient data were evaluated at week 28, week 108 and EOS. Although follow up was planned Q2W, a visit was not required if drug administration or assessment at the study site was not scheduled. The study protocol was approved by the institutional review board at each study site. The study was performed in compliance with the Declaration of Helsinki, and written informed consent was obtained from all patients.

### Patients

Patients aged 18 years or more with plaque psoriasis (psoriasis vulgaris with/without psoriatic arthritis) who completed 64 weeks of treatment with brodalumab were enrolled in the study. Patients were required to be hepatitis B negative at and after 48 weeks of brodalumab treatment, with no radiographic or computed tomography findings indicative of tuberculosis at week 52 of the previous study. Women of child‐bearing potential were required to have a negative urine pregnancy test result on day 1. Patients were switched to the post‐approval phase study at the physician’s discretion after obtaining their written informed consent. Exclusion criteria were as follows: serious infections requiring systemic antibiotic or antiviral therapy (excluding oral administration); non‐compliance with the study protocol; suicidal ideation severity 4 or 5, or suicidal behavior of any type while on treatment with brodalumab in the previous studies,^9^, ^10^ as determined by the Columbia – Suicide Severity Rating Scale (C‐SSRS) score on day 1; mental disorder or a history of mental disorder; severe depression (Patient Health Questionnaire 8 [PHQ‐8] depression scale score, ≥15) on day 1; and unfit for enrollment according to the physician. Women (of child‐bearing potential) and fertile men who were unwilling to use a single, highly effective contraception method or a combination of two contraceptive methods from the date of consent (if women) or the start of administration (if men) until 12 weeks after the end of administration were also excluded.

### Treatment

Subcutaneous treatment with brodalumab was initiated at a dose of 140 mg Q4W on day 1. Thereafter, two treatment strategies were implemented. Between weeks 2 and 28, the dose was either maintained or the dosing interval was shortened (140 mg Q4W → 140 mg Q2W), the dose directly escalated (140 mg Q4W → 210 mg Q2W) or both in a stepwise manner (140 mg Q4W → 140 mg Q2W → 210 mg Q2W) by using the static Physician’s Global Assessment (sPGA) score (either ≥3 [moderate or higher severity] or remains/is maintained at 2 [mild] for ≥4 weeks) at the physician’s discretion (rule 1). Eligible patients were switched to the Q2W dosing interval on an even week and further dose modification was allowed at the physician’s discretion; however, reverting to the Q4W dosing interval was not allowed. After week 28, in addition to an increase in the dose and dosing interval, a reduction in the dose and dosing interval only at the physician’s discretion even without an sPGA score was also allowed until drug approval (≥28 weeks; rule 2). However, when reducing the dose or extending the dosing interval, the following order was applied: 210 mg Q2W → 140 mg Q2W → 140 mg Q4W → 140 mg once every 8 weeks (Q8W). Finally, all patients were switched to brodalumab 210 mg Q2W after drug approval (Fig. S1).

Use of the following treatments was prohibited: phototherapy; topical therapy with calcineurin inhibitors, active vitamin D or A analogs and steroids; systemic therapy with salazosulfapyridine, azathioprine, thioguanine, hydroxyurea, fumaric acid esters, corticosteroids, calcineurin inhibitors (cyclosporin and tacrolimus), methotrexate and vitamin D or A analogs; coal tar therapy; biologics (adalimumab, infliximab, ustekinumab and etanercept) and live vaccines; and investigational drugs or devices. After week 28, topical therapy for psoriasis and systemic therapy as long‐term corticosteroid therapy (14 consecutive days) for comorbid diseases other than psoriasis were permitted.

### Assessments

#### Efficacy assessments

The Psoriasis Area and Severity Index (PASI) scores were evaluated at baseline (week 64) in the phase II study,^9^ on day 1 (week 52 of the previous study was considered as week 0), Q4W until week 28, and Q8W thereafter until week 108 or the week of discontinuation in the current extension study. Patients with psoriatic arthritis were evaluated using the American College of Rheumatology (ACR) response criteria for 20% (ACR 20), 50% (ACR 50) and 70% (ACR 70) improvement in the tender joint count on day 1, week 12, week 28, and every 16 weeks thereafter until week 108 or the week of discontinuation. The percentage of body surface area (BSA) involvement was evaluated at baseline in the phase II study, day 1, Q4W until week 28, and Q8W thereafter until week 108 or the week of discontinuation. Other efficacy parameters, such as the Nail Psoriasis Severity Index (NAPSI), Psoriasis Scalp Severity Index (PSSI) and Dermatology Life Quality Index (DLQI; patient‐reported outcome), were evaluated at baseline in the phase II study, day 1, week 12, week 28, and every 16 weeks thereafter until week 108 or the week of discontinuation.

#### Safety assessments

All adverse events (AE), including treatment‐related AE (TRAE), were recorded throughout the study period. Other serious TRAE were defined as serious TRAE with an outcome other than death, and other significant TRAE were defined as all non‐serious TRAE that led to withdrawal or an interruption or reduction in the dose of administration. Other safety assessments, such as the C‐SSRS and PHQ‐8 scores,^14^ were evaluated on day 1 and Q2W thereafter until week 108 or the week of discontinuation. These assessments were also performed Q4W throughout the study period at the physician’s discretion.

#### Anti‐brodalumab antibody evaluation

The incidence of anti‐brodalumab‐binding antibodies was evaluated at weeks 12 and 28, every 16 weeks after 28 weeks, and at the week of discontinuation. Anti‐brodalumab antibodies were measured using an electrochemiluminescent immunoassay (PPD, Wilmington, NC, USA). Samples positive for anti‐brodalumab‐binding antibodies were subsequently evaluated for anti‐brodalumab‐neutralizing antibodies by using a cell‐based bioassay (PPD; and Amgen, Thousand Oaks, CA, USA).

### Statistical analyses

Efficacy and safety analyses were performed for all patients except those with missing data. All end‐points were evaluated using the full analysis set. All efficacy assessments, including the patient‐reported outcomes, are summarized descriptively.

Categorical data are summarized as frequencies and percentages, and continuous variables are provided as summary statistics (number of patients, median [quartile 1, quartile 3], minimum and maximum). For patients who transitioned to the phase III study, “baseline” was defined as the time point before brodalumab initiation in the phase II study. The primary analysis was conducted using an intent‐to‐treat approach based on the actual brodalumab dose administered. Demographics and clinical characteristics are summarized for baseline and day 1. Quartiles of PASI and DLQI scores were calculated for each pre‐dose at week 28, week 108 and EOS after week 108. Quartiles for PASI, BSA, PSSI, NAPSI and DLQI were calculated, and frequencies for ACR 20, ACR 50 and ACR 70 were also determined. The quartiles for the PASI, DLQI, NAPSI and PSSI scores, and the numbers of patients for ACR 20, 50 and 70, were calculated and illustrated using a transition chart at baseline (except for ACR evaluation), on day 1, and at weeks 28 and 108. The number of relevant cases was also calculated for each time point and each pre‐dose. TRAE are presented according to the system organ class (SOC) and preferred term (PT) in the Medical Dictionary for Regulatory Activities – Japanese translation (MedDRA/J) version 17.1. Serious and other important TRAE were also tabulated similarly. PHQ‐8 assessments were extracted at the time of the initial survey (depending on the case, from day 1 to week 16). Numerical variables were calculated as quartiles, and categorical variables were calculated as frequencies and ratios. Changes in PHQ‐8 at all measurement time points were distributed according to the severity. The frequency and ratio of the C‐SSRS scores at all measurement time points were tabulated. All analyses were performed for patients without any missing data (i.e. list‐wise deletion or complete case analysis) using SAS software version 9.2 or 9.3 (SAS Institute, Cary, NC, USA).

## Results

### Patient disposition and baseline characteristics

**Figure 1:**
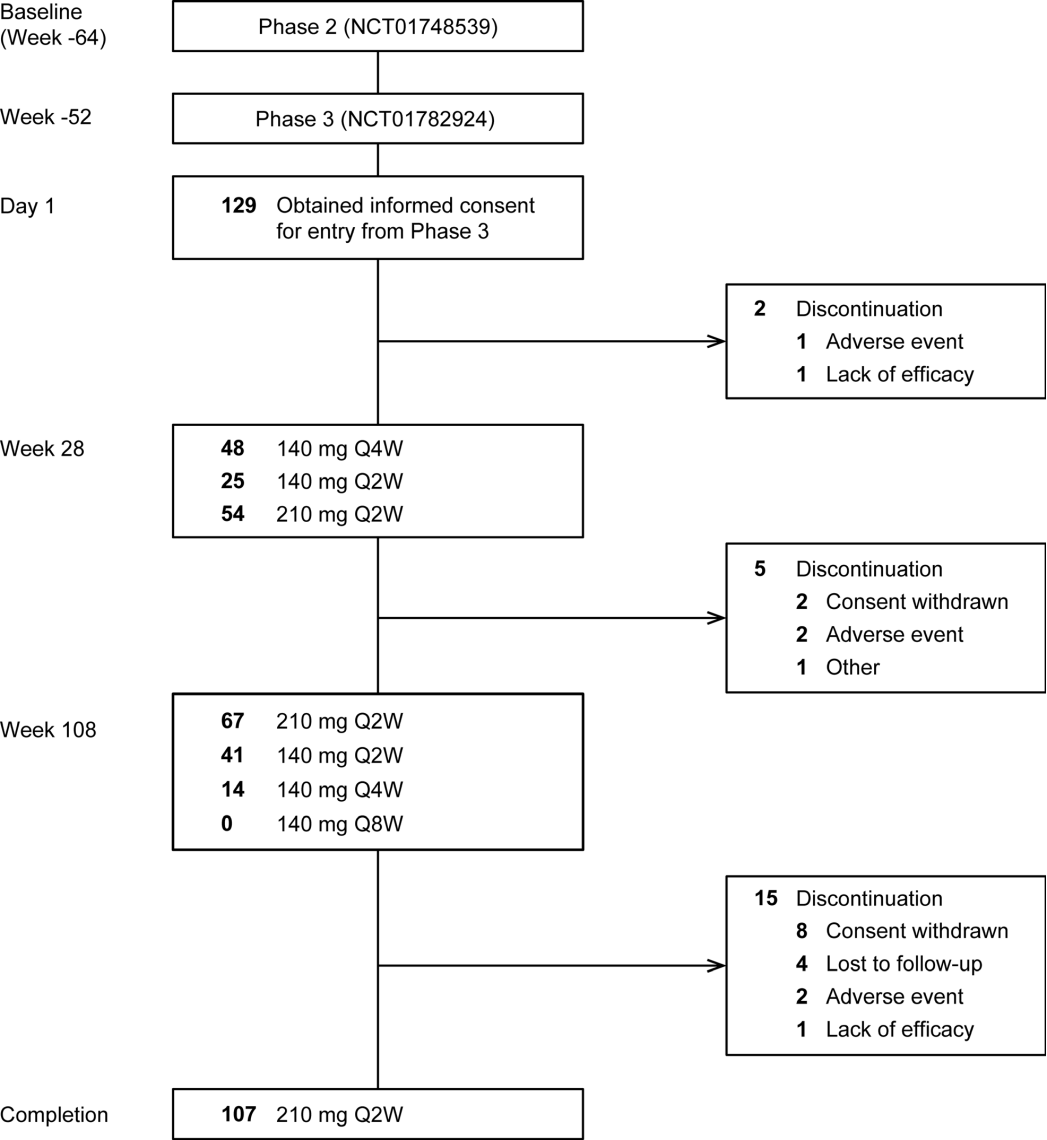
Patient disposition. Q2W, every 2 weeks; Q4W, every 4 weeks.

A total of 129 patients were enrolled, and all were switched to brodalumab 140 mg Q4W on day 1 (week 0). Of these, 107 (82.9%) patients completed the 108‐week or more follow up (EOS) (Fig. 1). Seven patients discontinued or withdrew from treatment before week 108 because of AE (*n* = 3: at week 15 [140 mg Q4W], week 72 [210 mg Q2W] and week 78 [140 mg Q2W]), lack of efficacy (*n* = 1: at week 20 [210 mg Q2W]), withdrawal of consent (*n* = 2: at week 42 [140 mg Q4W] and week 104 [210 mg Q2W]) and other (*n* = 1: at week 100 [140 mg Q4W]; resection for colorectal cancer during the treatment period). Fifteen patients discontinued/withdrew from treatment between 108 or more and 140 weeks or less because of AE (*n* = 2: at week 117 [210 mg Q2W] and week 130 [210 mg Q2W]), lack of efficacy (*n* = 1: at week 123 [210 mg Q2W]) and withdrawal of consent or lost to follow up (*n* = 11: 210 mg Q2W; and *n* = 1: 140 mg Q4W). The median (interquartile range [IQR]) age of the patients was 43.0 years (37.0–55.0) and 80.6% were men. All patients enrolled in the study had well‐controlled psoriasis (median [IQR] PASI score, 0 [0.0–1.2]), with none to a small effect on HRQoL (median [IQR] DLQI score, 1 [0.0–2]) on day 1 (week 0) (Table 1).

**Table 1:** Demographics and clinical characteristics at baseline and day 1

	Baseline	Day 1
Age, years	43.0 (37.0–55.0)	–
Sex		
Male	104 (80.6)	–
Female	25 (19.4)	–
Weight, kg	72.0 (62.5–84.5)	69.5 (60.9–83.8)
Body mass index, kg/m^2^	25.9 (22.9–28.7)	25.2 (22.7–28.8)
Duration of psoriasis, years	12.3 (6.2–20.2)	–
Body surface area, %	36.0 (23.0–52.0)	0.0 (0.0–2.0)
PASI score		
Total	24.7 (18.0–32.4)	0.00 (0.00–1.20)
210 mg Q2W	–	0.00 (0.00–0.90)
140 mg Q2W	–	0.05 (0.00–2.10)
DLQI		
Total	8.5 (5.0–15.0)	1.0 (0.0–2.0)
210 mg Q2W	–	0.0 (0.0–1.0)
140 mg Q2W	–	1.0 (0.0–2.0)
PHQ‐8^†^	–	1.0 (0.0–3.0)
Psoriatic arthritis	–	17 (13.2)
ACR 20, %	–	52.9
ACR 50, %	–	35.3
ACR 70, %	–	35.3
Nail psoriasis	–	81 (62.8)
NAPSI score	7.0 (4.0–10.0)	0.0 (0.0–2.0)
Scalp psoriasis	–	121 (93.8)
PSSI score	25.0 (12.0–36.0)	0.0 (0.0–2.0)

^†^ First administration time point after day 1 (day 1–week 16). Data are presented as *n* (%) or median (interquartile range). Each dose shows the last dose before day 1. ACR, American College of Rheumatology; DLQI, Dermatology Life Quality Index; NAPSI, Nail Psoriasis Severity Index; PASI, Psoriasis Area and Severity Index; PHQ‐8, Patient Health Questionnaire‐8; PSSI, Psoriasis Scalp Severity Index; Q2W, every 2 weeks.

### Efficacy

**Figure 2:**
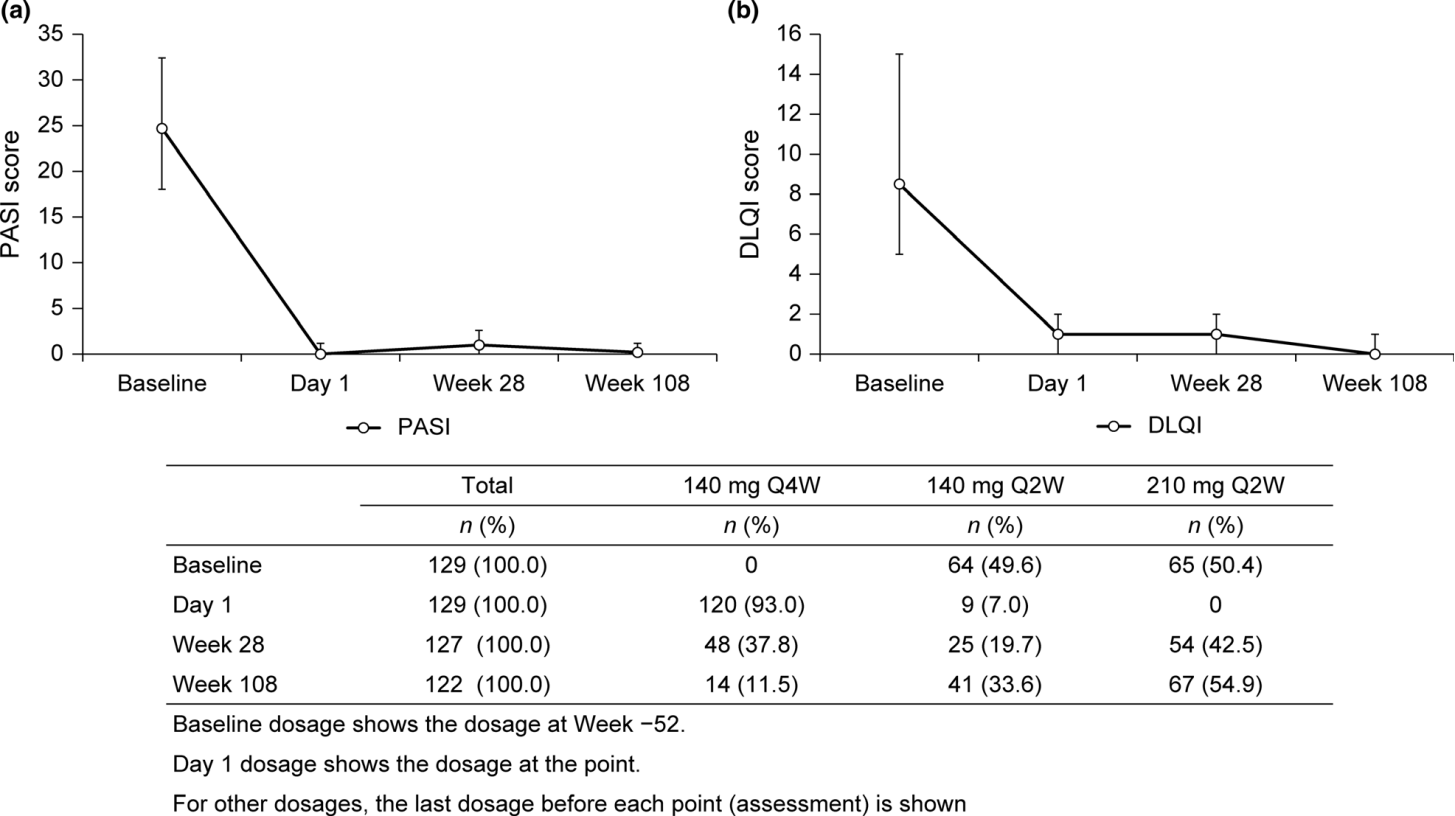
Median (IQR) (a) PASI and (b) DLQI scores and patient distribution of brodalumab dose and dosing interval at baseline, day 1, week 28 and week 108. DLQI, Dermatology Life Quality Index; IQR, interquartile range; PASI, Psoriasis Area and Severity Index; Q2W, every 2 weeks; Q4W, every 4 weeks.

At week 28, 54 of 127 (42.5%) patients were escalated to brodalumab 210 mg Q2W and 48 of 127 (37.8%) patients continued to receive brodalumab 140 mg Q4W; the dosing interval was shortened to 140 mg Q2W in 25 of 127 (19.7%) patients. Thereafter, at week 108, 67 of 122 (54.9%) patients were escalated to brodalumab 210 mg Q2W, and 14 of 122 (11.5%) patients remained on the initial dose of brodalumab 140 mg Q4W. Overall, 107 of 129 (82.95%) patients continued brodalumab treatment beyond 108 weeks, with the dose modified to 210 mg Q2W. Median (IQR) PASI scores and DLQI scores were low throughout the follow‐up period (week 28, week 108 and EOS) (Table 2, Fig. 2).

**Table 2:** PASI and DLQI scores at week 28, week 108 and EOS

Pre‐dose	*n*	PASI score	DLQI
**Week 28**			
Total	127	1.00 (0.00–2.60)	1.0 (0.0–2.0)
210 mg Q2W	54	0.85 (0.00–2.40)	1.0 (0.0–2.0)
140 mg Q2W	25	1.20 (0.00–2.40)	1.0 (0.0–3.0)
140 mg Q4W	48	1.20 (0.00–2.70)	1.0 (0.0–1.0)
**Week 108**			
Total	122	0.20 (0.00–1.20)	0.0 (0.0–1.0)
210 mg Q2W	67	0.20 (0.00–1.50)	0.0 (0.0–1.0)
140 mg Q2W	41	0.20 (0.00–1.10)	0.0 (0.0–1.0)
140 mg Q4W	14	0.55 (0.00–1.40)	0.0 (0.0–1.0)
**EOS after week 108**			
Total	107	0.00 (0.00–0.50)	0.0 (0.0–1.0)
210 mg Q2W	107	0.00 (0.00–0.50)	0.0 (0.0–1.0)

Each dose shows the last dose before each point. Data are presented as numbers or median (interquartile range). DLQI, Dermatology Life Quality Index; EOS, end of study; PASI, Psoriasis Area and Severity Index; Q2W, every 2 weeks; Q4W, every 4 weeks.

At EOS (range, 12–148 weeks), the severity of psoriasis was controlled in most patients, as indicated by the median (IQR) PASI score (0.00 [0–0.80]), BSA (0.00% [0–1.10]), PSSI score (0.0 [0–1.0]), NAPSI score (0.0 [0–2.0]), DLQI score (0.00 [0–1.0]) and PHQ‐8 score (0.0 [0–2]). At EOS (range, 12–148 weeks), of the 17 patients with psoriatic arthritis, eight (47.1%), five (29.4%) and four (23.5%) achieved ACR 20, 50, and 70, respectively.

At week 28, 53 of 119 (44.5%) and 34 of 81 (42.0%) patients, respectively, with scalp and nail symptoms were also escalated to brodalumab 210 mg Q2W, whereas 41 of 119 (34.5%) and 30 of 81 (37.0%) patients, respectively, continued on brodalumab 140 mg Q4W. The dosing interval was shortened to 140 mg Q2W in 25 of 119 (21.0%) patients with scalp symptoms and in 17 of 81 (21.0%) patients with nail symptoms.

Thereafter, at week 108, 64 of 114 (56.1%) and 42 of 76 (55.3%) patients, respectively, with scalp and nail symptoms were escalated to brodalumab 210 mg Q2W, and nine of 114 (7.9%) and five of 76 (6.6%) patients, respectively, remained on the initial dose of brodalumab 140 mg Q4W. The dosing interval was shortened to 140 mg Q2W in 41 of 114 (36.0%) patients with scalp symptoms and 29 of 76 (38.2%) patients with nail symptoms.

**Figure 3:**
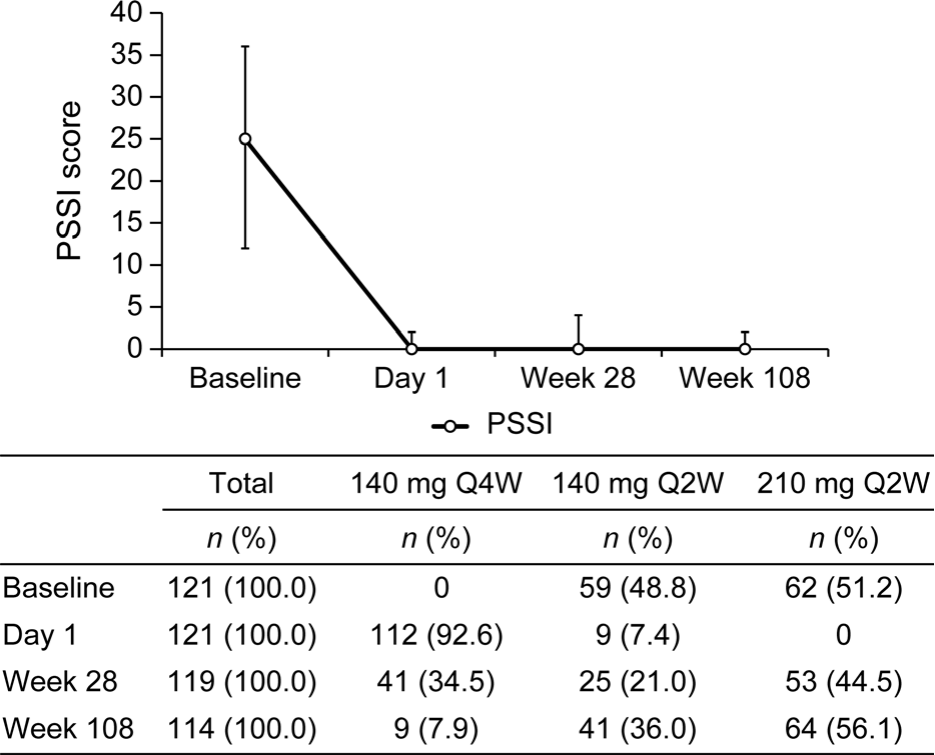
Median (IQR) PSSI scores and patient distribution of brodalumab dose and dosing interval at baseline, day 1, week 28 and week 108. IQR, interquartile range; PSSI, Psoriasis Scalp Severity Index; Q2W, every 2 weeks; Q4W, every 4 weeks.

**Figure 4:**
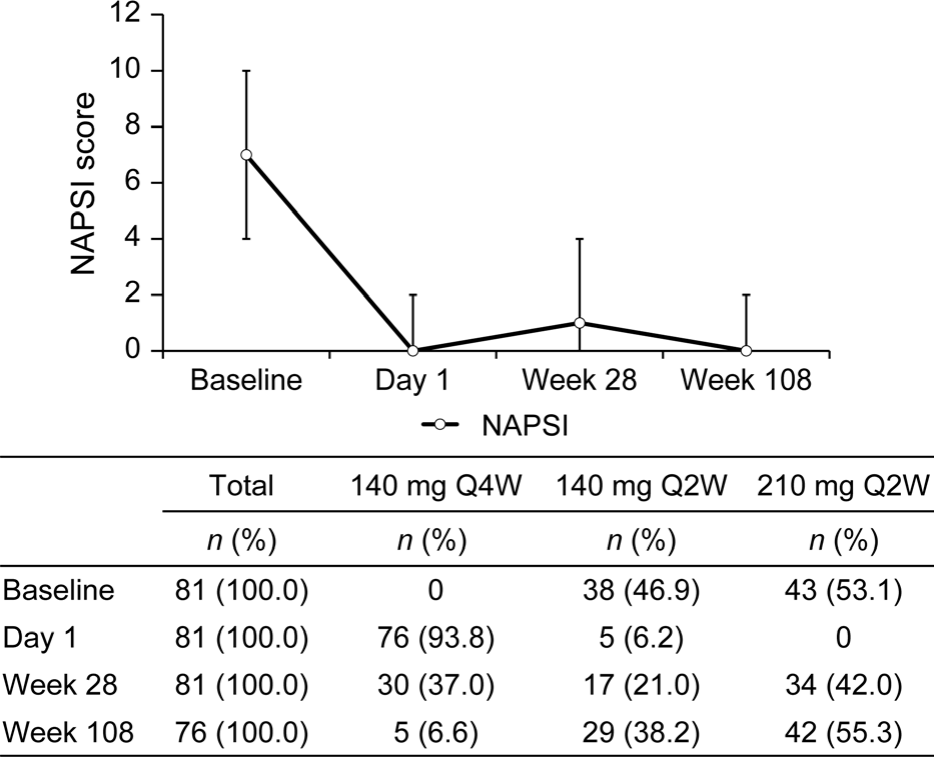
Median (IQR) NAPSI scores and patient distribution of brodalumab dose and dosing interval at baseline, day 1, week 28 and week 108. IQR, interquartile range; NAPSI, Nail Psoriasis Severity Index; Q2W, every 2 weeks; Q4W, every 4 weeks.

Median (IQR) PSSI scores remained low throughout the 108‐week follow‐up period (Fig. 3). The median (IQR) NAPSI score tended to increase at week 28 but remained low at week 108 (Fig. 4).

At week 28, nine of 17 (52.9%) patients with joint symptoms (ACR criteria) were escalated to brodalumab 210 mg Q2W and six of 17 (35.3%) patients continued to receive brodalumab 140 mg Q4W; the dosing interval was shortened to 140 mg Q2W in two of 17 (11.8%) patients.

**Figure 5:**
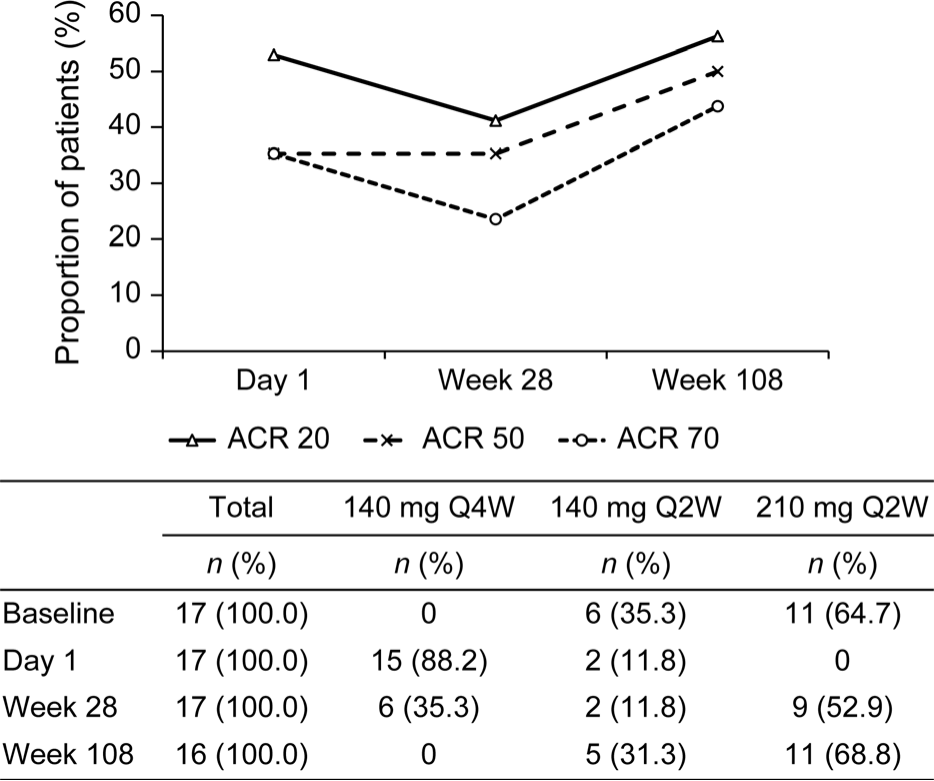
Proportion of patients achieving ACR 20, ACR 50 and ACR 70 and patient distribution of brodalumab dose and dosing interval at baseline, day 1, week 28 and week 108. ACR, American College of Rheumatology; Q2W, every 2 weeks; Q4W, every 4 weeks.

At week 108, 11 of 16 (68.8%) patients were escalated to brodalumab 210 mg Q2W and none of the patients remained on the initial dose of brodalumab 140 mg Q4W; the dosing interval was shortened to 140 mg Q2W in five of 16 (31.3%) patients. Compared with day 1, the proportion of patients who achieved ACR 20 and 70 tended to decrease at week 28; in contrast, the proportion of patients who achieved ACR 50 showed a consistent increase. Thereafter, the proportion of patients who achieved ACR 20, ACR 50 and ACR 70 showed an increase at week 108 (Fig. 5).

### Safety

Any TRAE were observed in 83 of 129 (64.3%) patients between day 1 and the EOS evaluation. The most commonly reported TRAE was nasopharyngitis (29/129 [22.5%]), followed by influenza and oral candidiasis (9/129 [7.0%] each), and folliculitis, cellulitis, pharyngitis, arthralgia, skin papilloma, periodontitis, diarrhea, upper respiratory tract inflammation and pyrexia (all reported in ≥3% of patients) (Table 3).

**Table 3:** Summary of treatment‐related adverse events (≥3% of patients; safety analysis population)

PT	Treatment‐related adverse events in patients (*n* = 129)	
	*n*	(%)
All	83	64.3
Nasopharyngitis	29	22.5
Influenza	9	7.0
Folliculitis	8	6.2
Oral candidiasis	9	7.0
Cellulitis	5	3.9
Pharyngitis	5	3.9
Arthralgia	5	3.9
Skin papilloma	5	3.9
Periodontitis	4	3.1
Diarrhea	4	3.1
Upper respiratory tract inflammation	4	3.1
Pyrexia	4	3.1

PT, preferred term.

No deaths were reported. Serious TRAE were observed in four (3.1%) patients and other significant TRAE in 15 (11.6%) patients. Serious TRAE, such as appendicitis, brain abscess, bacterial meningitis, colon cancer, IgA nephropathy and tubulointerstitial nephritis, were reported in one patient each. No cases of serious candida infection or Crohn’s disease were observed (Table 4). Treatment discontinuation due to AE was reported in five patients: one case before week 28, two cases between weeks 28 and 108, and two cases between week 108 and EOS (Fig. 1).

**Table 4:** Proportion of patients with treatment‐related adverse events (other serious or other significant) by PT (safety analysis population)

SOC	PT	*n* = 129	
		*n*	(%)
All		83	64.3
Death		0	
Other serious		4	3.1
	Appendicitis	1	0.78
	Brain abscess	1	0.78
	Meningitis bacterial	1	0.78
	Colon cancer	1	0.78
	IgA nephropathy	1	0.78
	Tubulointerstitial nephritis	1	0.78
Other significant		15	11.6
	Nasopharyngitis	3	2.3
	Influenza	1	0.78
	Cellulitis	1	0.78
	Folliculitis	1	0.78
	Pharyngitis	1	0.78
	Pyelonephritis	1	0.78
	Subcutaneous abscess	1	0.78
	Pneumonia bacterial	1	0.78
	Lung infection	1	0.78
	Upper respiratory tract inflammation	1	0.78
	Asthma	1	0.78
	Interstitial lung disease	1	0.78
	Pleural effusion	1	0.78
	Hepatic function abnormal	2	1.6
	Neutropenia	1	0.78

IgA, immunoglobulin A; PT, preferred term; SOC, system organ class.

**Figure 6:**
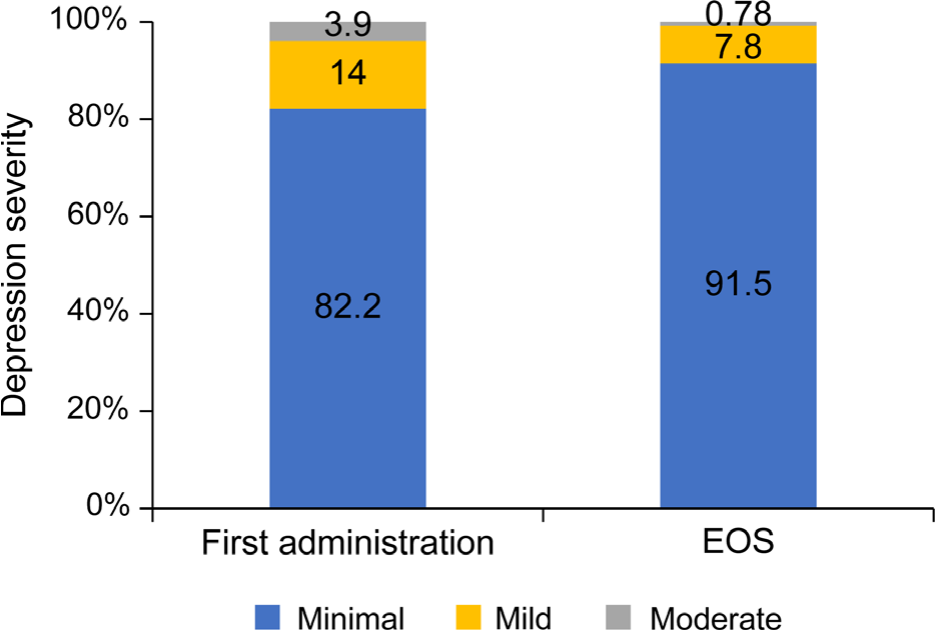
Change in PHQ‐8 score from the first administration of brodalumab to EOS. EOS, end of study; PHQ‐8, Patient Health Questionnaire 8.

The maximum suicidal ideation score (C‐SSRS) of the patients was 0 throughout the study period, except for two patients (data not shown). The proportion of patients with mild to moderate depression rated using PHQ‐8 did not increase throughout the study period (from treatment initiation to EOS); the PHQ‐8 score distribution was also similar among the patients (Fig. 6). No patient had a PHQ‐8 score of 15 points or more throughout the study period to the EOS evaluation. No anti‐brodalumab‐binding antibodies or brodalumab‐neutralizing antibodies were detected in any patient throughout the study period.

## Discussion

This OLE study demonstrated the safety and efficacy of long‐term brodalumab treatment in Japanese patients with plaque psoriasis who had completed the prior 64‐week study. Most patients completed the 108‐week extension study, and few patients discontinued because of AE or lack of efficacy during treatment, where the dose and dosing interval could be modified based on the severity of the disease at the physician’s discretion. Overall, 122 (94.6%) patients completed the 108‐week extension study, similar to 92% of patients who completed the previous 52‐week phase III study.^10^ Overall, no deaths were reported, and 3.1% of other serious TRAE and 11.6% of other significant TRAE were observed in this patient population. Of note, one patient who had a history of alcoholic liver dysfunction reported brain abscess and meningitis bacterial during the treatment of brodalumab at week 115. He was treated with antiprotozoal and antibiotics after the study discontinuation and recovered without further problems. No cases of newly diagnosed tuberculosis, severe candidiasis or Crohn’s disease (developed or reactivated) were observed throughout the study period.

The proportion of patients who continued brodalumab 140 mg Q4W in this study decreased to 37.8% at week 28 and 11.5% at week 108, despite the severity of psoriasis and HRQoL being well controlled throughout the study. It might have been necessary to escalate the dose or shorten the dosing interval considering the extent of worsening of the skin and scalp symptoms before evaluation, even if the PASI and PSSI scores were sufficiently low at each evaluation. Although the efficacy in patients with nail and joint symptoms tended to decrease temporarily at week 28, the severity of psoriasis and HRQoL were well controlled throughout the long‐term extension study.

On the other hand, this study showed that almost all patients with plaque psoriasis who completed 64 weeks of treatment had a median PASI score of 0 and a median DLQI score of 0 at EOS, and indicated that the effects of brodalumab may be sustained over the long term with the appropriate dose and dosing interval.

Brodalumab treatment was well tolerated throughout the long‐term extension study, and no new safety signals were identified. Although direct comparisons cannot be made because of differences in patients, follow‐up periods and brodalumab doses, the incidence and severity of AE were similar to those reported in previous clinical trials with brodalumab in psoriatic patients.^9^, ^10^, ^15^, ^16^, ^17^, ^18^ AE of interest that occur with IL‐17 inhibitors, such as serious candida infections or Crohn’s disease, were not observed in this study. Although this study did not enroll patients suspected to have or who were at risk of tuberculosis, we did not observe any new cases of tuberculosis in this study. By blocking critical mediators of adaptive and innate immunity, biotherapeutics may carry a risk of increased opportunistic infections. IL‐17A has a role in immune defense in mucocutaneous barrier tissues and may play a role during *Mycobacterium tuberculosis* infection.^19^

Furthermore, depressive symptoms based on PHQ‐8 distribution appeared not to change through the study period from the first administration of brodalumab to EOS. The association between brodalumab exposure and suicidal ideation and behavior has been controversial,^20^, ^21^ but in this OLE study, two patients had a C‐SSRS maximum suicidal ideation severity with no causal association: one patient reported severity 2 at the evaluation after the start of the study and the other patient reported severity 1 at week 16. Only one patient had suicidal ideation and behavior; however, both events were due to economic troubles and were deemed by the physicians as unrelated to brodalumab. Another patient had suicidal ideation at week 16. Specifically, the patient stated, “thought about it a little because of exacerbation of psoriasis” and responded by saying “no” to a question about the specificity of active suicidal ideation. The degree of the suicidal ideation was also of the least severity. Thus, it may be also considered inadequate to reduce the dose or extend the dosing intervals easily even in the case of shared decision‐making with patients.

Immunogenicity with brodalumab may be low. Although treatment discontinuation due to lack of efficacy was reported in two patients, we did not observe any anti‐brodalumab‐binding antibodies and anti‐brodalumab‐neutralizing antibodies in this patient population on long‐term brodalumab treatment at any assessment point. This observed low immunogenicity profile of brodalumab was consistent with that reported in previous clinical trials.^22^, ^23^

In this study, no new safety signals were identified, and no anti‐brodalumab‐binding antibodies were observed with prolonged treatment. The severity of psoriasis and HRQoL were well controlled throughout the study period with brodalumab. Overall, the long‐term efficacy and safety of brodalumab were demonstrated, and the severity of psoriasis and HRQoL were well controlled over 108 weeks of treatment with brodalumab.

### Limitations

This was a single‐arm study without a placebo arm for comparison of efficacy. The AE might have been underestimated because all patients did not receive the approved dose regimen of brodalumab. The baseline PHQ‐8 assessments were not evaluated; therefore, whether the PHQ‐8 scores changed because of the treatment with brodalumab remains unelucidated. The ACR has been evaluated in a limited number of patients with plaque psoriasis with arthritis, and their joint sites were not fully analyzed.

## Conflict of Interest

This study was funded by Kyowa Kirin. Y. Y. reports grants and/or speaker honoraria from Kyowa Kirin, Celgene, Janssen Pharmaceutical, AbbVie, Maruho, Mitsubishi. Tanabe Pharma and Eli Lilly Japan outside the submitted work. H. N. received consulting fees and/or speaker honoraria from AbbVie, Eisai, Eli Lilly Japan, Janssen, Japan Tobacco, Kyowa, Kirin, LEO Pharma, Maruho, Novartis, Torii Pharmaceutical and UCB Japan. N. T. and K. O. are employees of Kyowa Kirin.

## Supporting information


**Figure S1.** Study design. Q2W, every 2 weeks; Q4W, every 4 weeks; Q8W, every 8 weeks.
