# Supplementary figures and images for: Long‐term safety of brodalumab in Japanese patients with plaque psoriasis: An open‐label extension study
Source: J Dermatol. 2020 Apr 10;47(6):569–77. doi: 10.1111/1346-8138.15343 (PMC7318217; doi:10.1111/1346-8138.15343)

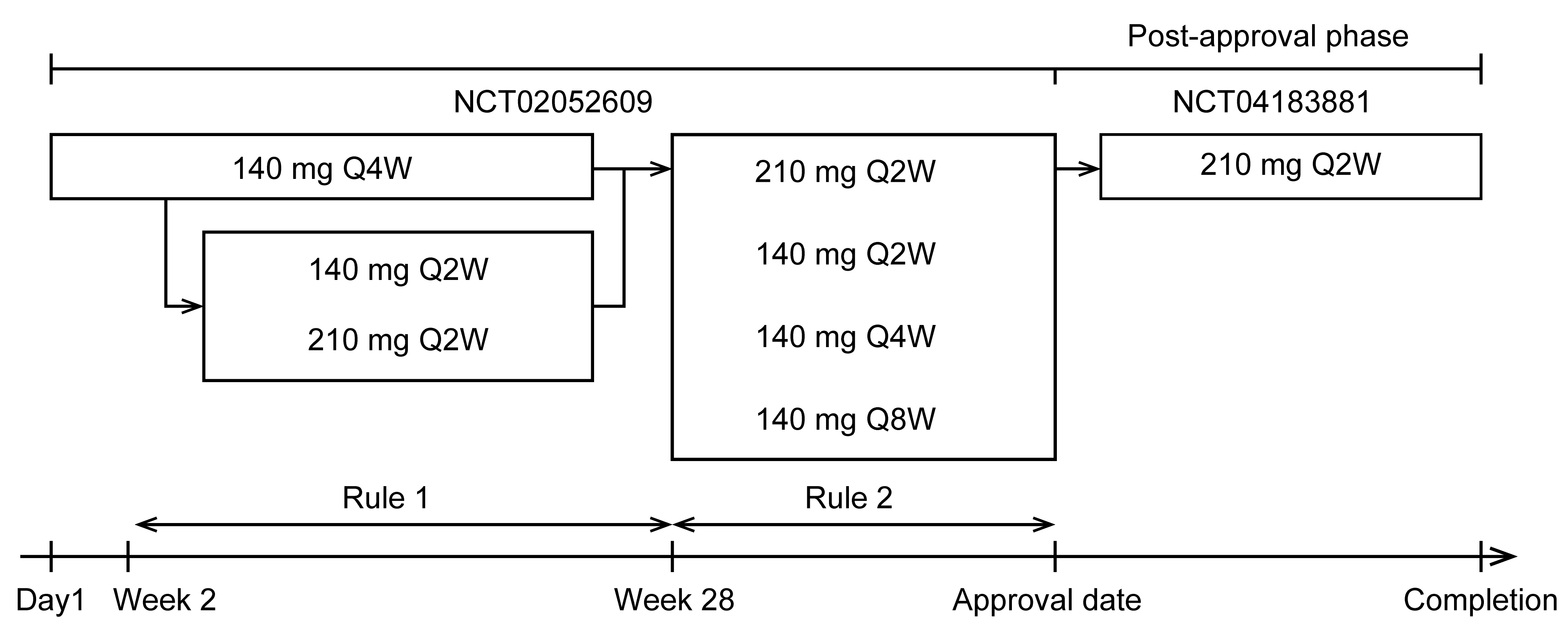

Supplement: Supplementary file 1 — Figure S1. Study design. Q2W, every 2 weeks; Q4W, every 4 weeks; Q8W, every 8 weeks. [file JDE-47-569-s001.tif]
